# Exploring Animal Models That Resemble Idiopathic Pulmonary Fibrosis

**DOI:** 10.3389/fmed.2017.00118

**Published:** 2017-07-28

**Authors:** Jun Tashiro, Gustavo A. Rubio, Andrew H. Limper, Kurt Williams, Sharon J. Elliot, Ioanna Ninou, Vassilis Aidinis, Argyrios Tzouvelekis, Marilyn K. Glassberg

**Affiliations:** ^1^Department of Surgery, University of Miami Miller School of Medicine, Miami, FL, United States; ^2^Department of Medicine, Mayo Clinic College of Medicine, Rochester, MN, United States; ^3^Department Pathobiology and Diagnostic Investigations, College of Veterinary Medicine, Michigan State University, East Lansing, MI, United States; ^4^Division of Immunology, Biomedical Sciences Research Center “Alexander Fleming”, Athens, Greece; ^5^Department of Medicine, University of Miami Miller School of Medicine, Miami, FL, United States

**Keywords:** bleomycin, idiopathic pulmonary fibrosis, murine model, asbestosis, aged mice

## Abstract

Large multicenter clinical trials have led to two recently approved drugs for patients with idiopathic pulmonary fibrosis (IPF); yet, both of these therapies only slow disease progression and do not provide a definitive cure. Traditionally, preclinical trials have utilized mouse models of bleomycin (BLM)-induced pulmonary fibrosis—though several limitations prevent direct translation to human IPF. Spontaneous pulmonary fibrosis occurs in other animal species, including dogs, horses, donkeys, and cats. While the fibrotic lungs of these animals share many characteristics with lungs of patients with IPF, current veterinary classifications of fibrotic lung disease are not entirely equivalent. Additional studies that profile these examples of spontaneous fibroses in animals for similarities to human IPF should prove useful for both human and animal investigators. In the meantime, studies of BLM-induced fibrosis in aged male mice remain the most clinically relevant model for preclinical study for human IPF. Addressing issues such as time course of treatment, animal size and characteristics, clinically irrelevant treatment endpoints, and reproducibility of therapeutic outcomes will improve the current status of preclinical studies. Elucidating the mechanisms responsible for the development of fibrosis and disrepair associated with aging through a collaborative approach between researchers will promote the development of models that more accurately represent the realm of interstitial lung diseases in humans.

## Background

Idiopathic pulmonary fibrosis (IPF) is a devastating chronic lung disease, primarily affecting middle aged and older adults (1, 2). Lung function decline is gradual, with the potential for intermittent, unpredictable, acute exacerbations and the development of associated pulmonary hypertension (3). Disease diagnosis is primarily based on a typical radiology pattern (high-resolution computed tomography—HRCT) of usual interstitial pneumonia (UIP) characterized by reticulation and honeycomb cysts of subpleural and bibasilar distribution coupled with exclusion of other known causes of lung fibrosis as assessed by absence of exposures (occupational, environmental, drug), a negative immunologic profile and compatible bronchoalveolar lavage fluid (BALF) findings (i.e., absence of lymphocytosis). Hallmark features of UIP include epithelial cell hyperplasia, basement membrane denudation, honeycomb cysts, and accumulation of myofibroblasts foci in a pattern with regional and temporal heterogeneity (4).

Disease pathogenesis still remains elusive and controversial. Currently, the prevailing hypothesis assumes an ineffective wound healing response to alveolar epithelial cell injury (5, 6). Injury magnitude and susceptibility appears to be related to aging and genetic predisposition, with subsequent innate immune system and fibroblast activation (3, 5, 7). The overall prognosis of patients with IPF is highly unpredictable and poor with median survival after diagnosis being approximately 3.8 years (3, 8, 9). Attempts to understand disease pathogenesis, identify prognosticators, and unravel novel therapeutic targets (10–14) have relied on animal models. Unfortunately, no animal model fully recapitulates the histologic pattern of UIP or exhibits features of progressive disease. This, however, should not underestimate the fact that animal models are essential prerequisites for the subsequent application of prognostic tests and therapeutic interventions. Numerous clinical trials have been completed based on preclinical studies in animals and have led to the FDA approval of two drugs, pirfenidone and nintedanib (15, 16). Although these drugs slow disease progression, they do not cure IPF (5, 17), thus at the best case leaving patients with significant pulmonary disability. Therefore, further studies in animal models that more closely mimic human IPF are needed to investigate potentially curative therapies. Although it is recognized that the spontaneous development of lung fibrosis in domestic animals (cats, dogs, etc.) can be informative, the most indispensable models for studies of pathogenesis and preclinical therapeutic assessment involve rodents. Many traditional and newly developed experimental models have provided us with valuable insights into disease pathogenesis and helped us to identify novel therapeutic targets to assess and validate in clinical trials (18–20). For a tabular representation of these models, see Table 1. This review aims to summarize current state of knowledge on animal modeling of lung fibrosis, mainly focusing on rodents, including environmental and genetic models, highlight limitations, and suggest future potentials.

**Table 1:** Selected pulmonary fibrosis conditions in animal species.

Selected pulmonary fibrosis conditions in animal species			

Species	Model/disease	Features	Histology
Mouse (C57BL/6J)	Bleomycin (BLM) (experimental)	Increased collagen deposition	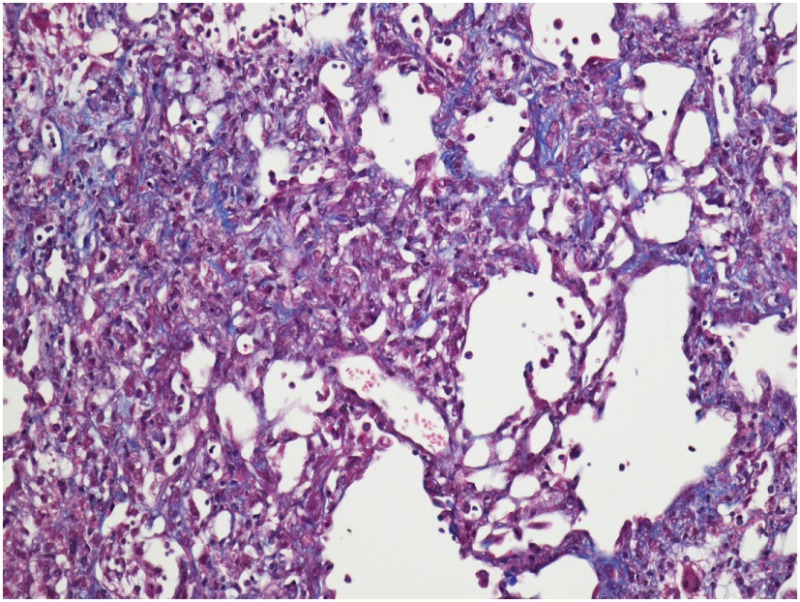
		Patchy fibrosis associated with inflammatory infiltrates	
		Resolution in young mice starting at 3 weeks	

Dog (West Highland Terrier)	Interstitial lung disease (ILD)	Septal widening and collagen deposition	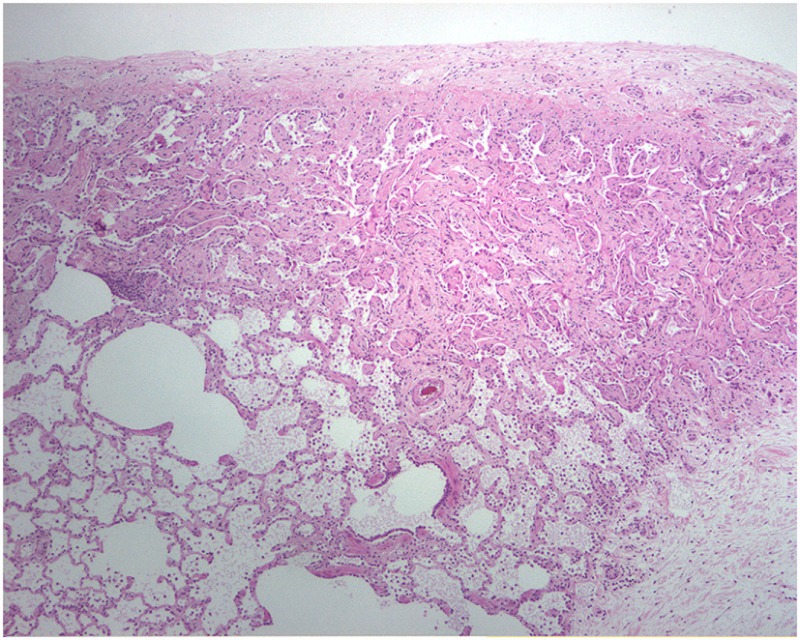
		Normal alveolar cells	

Donkey	Chronic pleuropulmonary fibrosis	Associated with asinine herpesvirus 5 infection	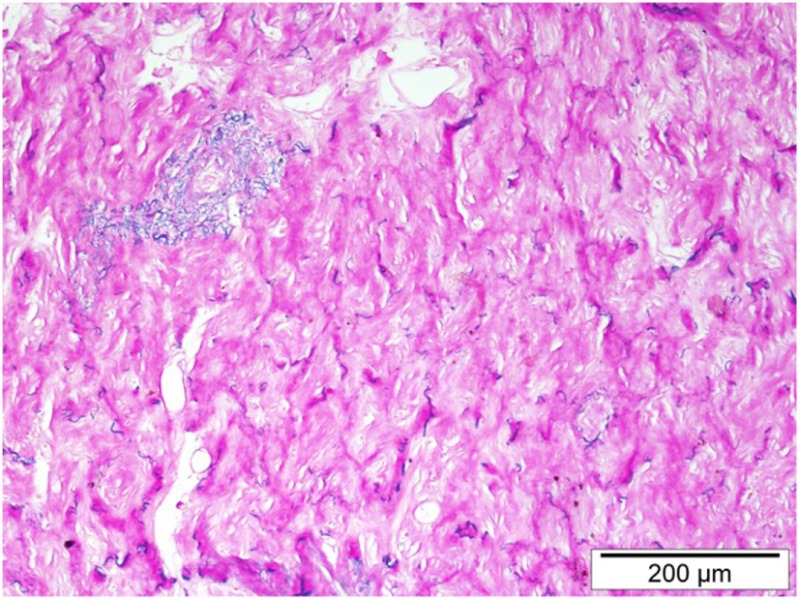
		Pleural, subpleural, and septal fibrosis extending to interstitium	
		Intra-alveolar fibrosis and alveolar septal elastosis	

Horses	Equine multinodular pulmonary fibrosis	Associated with equine herpesvirus 5 infection	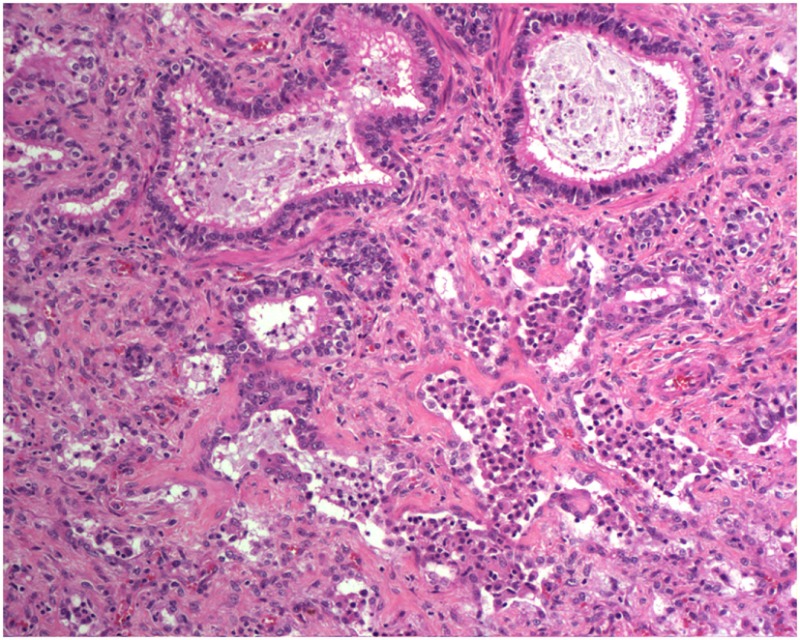
		Multifocal coalescing nodules within parenchyma, centered on alveoli	

Cats	Idiopathic pulmonary fibrosis	Temporally heterogeneous fibrosis without inflammation	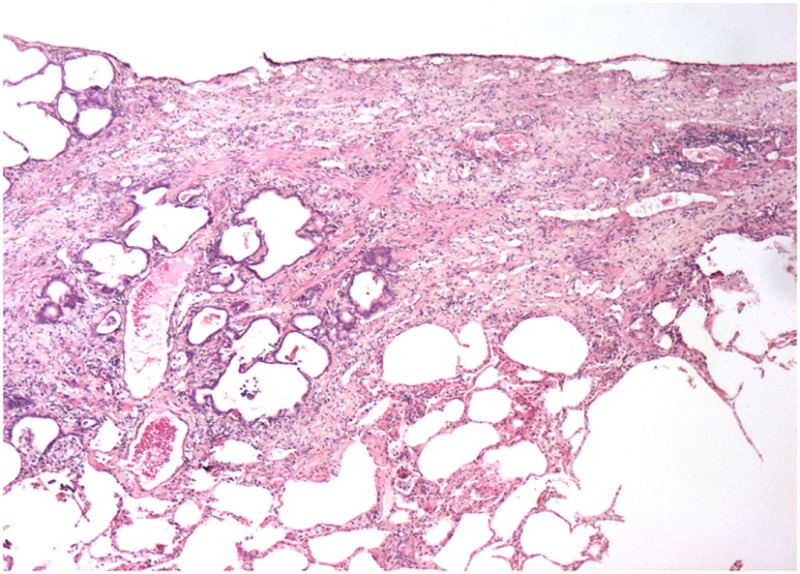
		Patchy remodeling leading to honeycomb lung in late disease	

*A variety of animal species exhibit pulmonary fibrosis conditions with characteristics similar to idiopathic pulmonary fibrosis (IPF) in humans. ILD has been studied as fibrosis occurs spontaneously in many animals, including West Highland Terriers, donkeys, cats, and horses. Mice have been used as experimental models with BLM-induced pulmonary fibrosis. Histologic images are provided at 10–20× magnification on Trichrome stain to display representative characteristics and similarities. Reproduced with permission, from Dr. Paul Mercer. *Clin Sci* (2015) 128:235–256, © The Biochemical Society (97)*.

## Murine Models

### Bleomycin (BLM)

The model of BLM-induced lung fibrosis represents the most commonly applied experimental model. BLM is a chemotherapeutic antibiotic that has been identified as a pro-fibrotic agent when lymphoma patients developed pulmonary fibrosis after intravenous administration of BLM. It has been used in multiple species including mice, rats, guinea pigs, hamsters, dogs, and primates; yet, mice are most common (21). A sheep model is also currently under development (22). A recent ATS workshop report confirmed that there is a consensus view of the intratracheal BLM model as “the best-characterized animal model available for preclinical testing” (23).

#### Mechanism of Action and Kinetics of Injury

It is believed that BLM acts by causing single and double-strand DNA breaks in tumor cells and thereby interrupting cell cycle leading to apoptosis. BLM hydrolase, a BLM-inactivating enzyme, majorly affects drug effects on a tissue-specific basis. The lungs maintain low levels of the enzyme, as compared to liver, and therefore are more susceptible to BLM-induced injury. An overproduction of reactive oxygen species, due to chelation of metal ions and reaction of the formed pseudoenzyme with oxygen, leads to epithelial cell death (days 1–3), excessive inflammatory infiltrates (days 3–9, neutrophils found in the BALF at day 3 and lymphocytes at day 6), and ultimately to fibroblasts activation, extracellular matrix deposition, and development of fibrosis (days 10–21 with a peak around day 14), at the molecular (24–26) and histologic (21, 24, 25, 27) levels. Relative to IPF, it has been shown that the early molecular signature in mice is most similar to the accelerated acute phase of IPF in humans (28). Measurements of alveolar septal thickening, intra-alveolar fibrosis, increases in alveolar macrophages, and dilation of bronchioles and alveolar ducts demonstrated a rather uniform fibrotic state in a large sample size (29). Nevertheless, BLM-induced lung fibrosis has been severely criticized for not being representative of IPF due to the rapidity of its development, inflammation preceding fibrosis, and self-resolution nature usually after 21–28 days following BLM challenge.

#### Strains, Gender, and Age

C57BL/6J mice have been the predominant animal model, as this particular strain is highly susceptible to lung injury following intratracheal BLM administration (30, 31). Conversely, the BALB/c or SV129 strains confer resistance to BLM-induced pulmonary fibrosis, presumably due to alterations in transforming growth factor (TGF)-β expression (31). This phenomenon parallels the experience in humans regarding genetic susceptibility and other potential risk factors for development of fibrosis in end organs following exposure to BLM. The majority of studies investigating BLM-induced pulmonary fibrosis to date have used young male mouse models, aged 8–12 weeks (28, 29, 32). Young mice, however, have been shown to undergo spontaneous resolution of BLM-induced pulmonary fibrosis, a phenomenon not observed in aged mice (24, 33, 34). Whether sex differences in mice parallel human IPF, which exhibits a tendency toward male predominance has not been fully determined. However, the use of aged male mice may provide a more clinically relevant model of IPF (33).

#### Route of Delivery and Dose Regimens

So far, BLM has been delivered by multiple methods including intratracheal, intraperitoneal, subcutaneous, intravenous, and inhalational. However, intratracheal is the most commonly route of administration (21, 24, 26, 28, 29, 32, 35–39). It is believed that intratracheal administration better recapitulates the human phenotype that is limited to the lungs. However, it requires a surgical incision at the level of the trachea, and thus, it is associated with considerable peri-operative mortality. To this end, investigators are now applying the orotracheal route of delivery that exhibits similar kinetics of injury as intratracheal administration with significantly less side effects.

Another issue identified in studies using the BLM mouse model is the wide range of dosing regimens used (40). In mouse studies, weight-based dosing is most common, beginning at 1.25 U/kg (39) and up to a maximum of 4 U/kg (35, 36). This dose is usually suspended in 50–100 µL of phosphate-buffered saline for intratracheal instillation. Peng et al. performed BLM dose-escalation experiments with mortality rates of 19% with 3 U/kg and 50% with 5 U/kg (28). A slightly lower dose of 2.0–2.5 U/kg appears to provide the most effective model of lung fibrosis, while reducing sample loss due to high mortality. With regard to frequency of dosing, Degryse and colleagues directly addressed the issue of single versus repetitive dosing to model IPF using BLM-induced pulmonary fibrosis (24). Results from their investigation in young mice found that repetitive dosing of BLM promoted persistent fibrosis, evidenced by measures of hydroxyproline content and inflammatory cell infiltrates, in contrast to single dose experiments that demonstrated spontaneous resolution in young mice (24, 34). Most studies evaluating therapeutic interventions have not used repetitive dosing; rather, the use of a single dose of intratracheal BLM is usually followed shortly by administration of the therapy under investigation (35–38). Potential therapies are usually administered within 1–7 days following BLM exposure, leading to the conclusion that the therapeutic measures may provide benefit primarily through prevention of the inflammatory cascade rather than reversal of fibrosis, thus limiting their applicability to human IPF (40). More recent studies have begun to explore administration of drugs after 7 days (41, 42). To our knowledge, only two studies to date have evaluated repetitive BLM injury (43, 44). Lee et al. administered intratracheal BLM (0.04 U) biweekly for a total of 4 months in young mice (43). They reported that in response to repeated BLM administration, mice developed hyperplasia of Club cells (Clara cells) and cuboidal alveolar epithelial cells, infiltration of the perialveolar ducts by inflammatory cells, septal thickening, enlarged alveoli, and extensive fibrosis (43).

### Silica

Silica administration into murine lungs leads to the development of fibrotic nodules that resemble lesions that develop in humans following exposure to mineral fibers and particulate aerosols (45). Silica delivery presents with many variations including aerosolization, intratracheal, or orotracheal instillation (46–50). The fibrotic response is strain dependent with C57BL/6 mice found to be more susceptible than CBA/J mice after intratracheal delivery of silica fibers. Nodules develop around silica deposits and silica fibers are easily identified both by histology and polarization microscopy (47). The fibrotic response is associated with limited inflammation and enhanced fibrotic lesions mediated by increased production of pro-fibrotic growth factors and cytokines including PDGF, TGF-β, TNFa, and IL-10 (51–53). Kinetics of injury is highly heterogeneous and dependent upon route of administration, dose regimens, and formulations of silica particles (45). In particular, intratracheal models are easier, shorter (fibrosis develops within 14–28 days), and cost-efficient, while aerosolized route of delivery takes longer to produce fibrotic lesions (40–120 days) (45). Heating preparation procedures before instillation are mandatory in order to inactivate any trace endotoxin (45). The greatest advantage of the silica model of lung fibrosis is the persistence of fibrotic lesions due to diminished clearance of silica particles from the lungs (51, 52, 54). However, the model presents with major caveats including problematic and highly expensive equipment for aerosolized delivery, prolonged waiting periods until development of fibrosis (4–16 weeks), lack of reproducibility of fibrotic pattern, and absence of characteristic UIP-like lesions such as fibroblastic foci, regional heterogeneity, and hyperplastic epithelium. These have severely limited its widespread applicability in the preclinical setting (20).

### Asbestosis

Another model that recapitulates an important form of human lung fibrosis is that of asbestos exposure. Asbestos-induced model of lung fibrosis is clearly distinguished from IPF by several histologic findings including asbestos bodies embedded within the fibrous tissue, fewer myofibroblasts foci and bronchial wall fibrosis. In some cases, the pattern of UIP can be also present (55, 56). Some of these features are recapitulated in inhalation models in animals and have helped us understand the pathogenesis of both asbestosis and IPF (57–61). A single intratracheal administration of asbestos fibers mediates development of fibrosis; however, the model presents with several caveats since fibrosis that tends to be central rather than subpleural and is quite often unevenly distributed between lungs. Inhalation models develop a more peripheral pattern; yet, disease development can be prolonged, especially if using chrysotile fibers. The intratracheal animal models with amphibole fibers follow the kinetics of BLM models, with fibrosis development at day 7 and peak at day 14. Inhalation models may take up to a month for establishment of fibrotic injuries. Mechanistically, the deposition of asbestos fibers triggers fibrosis through alveolar epithelial cell apoptosis, M2 polarization of macrophages, and overproduction of pro-fibrotic cytokines by activated T lymphocytes, all events leading to myofibroblast differentiation and extracellular matrix production (57–61).

### Age-Related Models

IPF is an age-related disease paradigm, and more recently, it has been proposed that many of the hallmarks of aging including genomic instability, telomere attrition, epigenetic alterations, deregulated cellular bioenergetics, and cellular senescence, can be considered characteristics of the fibrotic lung (62–64). Studies have shown that older mice are more susceptible than younger mice to pro-fibrotic stimuli including BLM (26). This is of particular interest given that IPF is predominant in older individuals. Transgenic deletion of senescence-related genes including RAGE, and relaxin has been associated with spontaneous age-dependent development of lung fibrosis indicating the cardinal role of aging in disease susceptibility (65–67). On the other hand, the role of “virome” as a pro-fibrotic mediator has been further dissected in the context of aged-related development of lung fibrosis by demonstrating that only aged mice (>15 months) develop γ-herpesvirus-68-induced lung fibrosis through a mechanism that involved alveolar epithelial cell reprogramming to produce pro-fibrotic factors and enhanced TGF-β signaling in lung fibroblasts (68). In addition, Torres-Gonzalez et al. (69) reported that aging mice receiving gamma herpesvirus responded with endoplasmic reticulum stress, apoptosis of type II lung epithelial cells, and activation of profibrotic pathways.

This evidence could be reminiscent to the presence of herpes viral genomes within IPF lungs and the epidemiological association between viral infections and IPF acute exacerbations (70).

### Cytokine Overexpression

During the past two decades, more sophisticated and advanced methods have been widely used to study the features of lung fibrosis on an experimental setting. Both gene transfer *via* adenoviral or lentiviral vectors and transgenic approaches have been used to overexpress pro-fibrotic cytokines including TGF-β, TNF-α, IL-1β, and IL-13 and promote fibrotic phenotypes by dissecting downstream signaling pathways that are highly relevant to human lung fibrosis (71–73). The overexpression of TGF-β can be produced via adenoviral intranasal delivery or doxycycline-induced transgenic overexpression in CC-10-positive lung epithelial cells. Both models are strain dependent with C57BL/6 mice being more susceptible that BALB/c. In the doxycycline-inducible Clara cell (CC10)-promoter driven model of TGF-β-induced lung fibrosis, addition of doxycycline to the water of animals leads to release of the tetracycline-controlled transcriptional suppressor allowing the reverse tetracycline transactivator to bind to the transgene (TGF-β) and promote its acute expression even 12 h after treatment with doxycycline (74). That leads to alveolar epithelial apoptosis and myofibroblast accumulation leading to airway and parenchymal fibrotic response starting at day 7 and peaking at days 14–21. Fibrosis may persist and progress over the duration of doxycycline exposure for up to 2 months (74). Similar kinetics are also observed with adenoviral delivery of TGF-β through the intranasal route leading to epithelial cell apoptosis (day 1), mononuclear cell infiltration (days 3–7), and fibrotic scarring that tends to be more persistent than those produced by BLM exposure and thus tend to mimic better human disease features (71). Nevertheless, both models quite often produce highly variable and heterogeneous kinetics of injury with regards to severity and extent of lesions and lack of major reproducibility. A similar concept has been also applied for adenoviral-mediated gene transfer of IL-1β (75) and TNF-α (76, 77) or lung-specific transgenic overexpression of IL-13 (78), thereby resulting in an early inflammatory response and later collagen deposition through activation of TGF-β signaling pathway. Nonetheless, these models are not well established and thus can only be used to dissect relevant pathogenic pathways and not on a general basis to recapitulate the complexity of human disease.

### Other Models

*Fluorescent isothiocyanate (FITC)* is another chemical compound used to induce experimental lung fibrosis (79). Fluorescein acts as a hapten and binds to airway proteins, thus acting as a depot for prolonged exposure to the injurious stimulus leading to fibrotic responses within 2–4 weeks that persist up to 24 weeks (79, 80). The model produces relatively reproducible and persistent fibrotic phenotypes in both BALB/c and C57BL/6 mice. Disadvantages include absence of representative UIP findings and predominant inflammatory infiltrates that precede fibrosis (80). The model is mostly dependent on Th2 cytokines (IL-13) and was seminally discovered to explore the relationship between the chemokine signaling receptor 2 (CCR2) and its ligand CCL12 for recruitment of fibrocytes during progression of fibrosis (81). It offers the advantage of easily trackable fluorescence-labeled fibrotic tissues. Nevertheless, model robustness is largely dependent on technical issues that are highly variable including the batch of FITC and the size of the particles formulated through sonication (20). Smaller particle sizes due to prolonged sonication may lead to acute toxicity and death (20). Finally and most important, FITC is an artificial chemical compound with limited human relevance since this type of injurious stimulus has never been described in humans (20).

*Radiation-induced fibrosis* represents a human relevant injury that leads to development of fibrosis which is strain dependent (C57BL/6 are the most susceptible) and can be local or systemic if other organs are not shielded (82–87). It is a relatively slow procedure that results in mature fibrosis after 24 weeks; yet, fibrosis is majorly dependent on inflammation and free-radical-mediated DNA damage and less on TGF-β (84).

*Familial models of IPF* have been also used to study the contribution of the disease genetic background that significantly altered our perspective regarding disease pathogenesis and treatment response. Mice with targeted deletion of shelterin, a six-protein complex that binds and preserves telomeric repeats, from type alveolar epithelial cells, have been shown to develop spontaneous fibrosis (88, 89).

Mutations in the telomere and telomerase genes have been associated with familial IPF (90). Telomere dysfunction results in alveolar epithelial stem cell senescence, which is sufficient to drive lung remodeling and recruit inflammation. Telomerase reverse transcriptase has been reported to be transiently increased in BLM, hypoxic, or silica-induced lung injury (91–93). On the other hand, telomerase-deficient mice, despite significant telomere shortening, did not present with enhanced BLM-induced fibrotic responses (94).

Although mutations resulting in SP-C deficiencies are linked to a small subset of spontaneous and familial cases of interstitial lung disease (ILD) and interstitial pulmonary fibrosis (95, 96), SP-C-deficient mice do not fully recapitulate familial interstitial pulmonary fibrosis (97) as they develop mild ILD and an emphysematous phenotype. It is more than evident that these mutations may generate a susceptible phenotype; yet, a second hit of environmental origin is needful to partially recapitulate human phenotype.

*Finally, humanized models of lung fibrosis* involving the intravenous instillation of human IPF lung fibroblasts into immunodeficient non-obese diabetic mice (NOD/SCID) have recently garnered much attention (98–100). This model allows for cell trafficking during different stages of fibrosis development and progression, offers unique insights into different fibroblast populations that reflect IPF heterogeneity, and dissects the contribution of epithelial–fibroblast crosstalk into the disease pathogenesis considering the absence of immune cells (99). Nevertheless, the latter is not representative of human disease where immune cells appear to play cardinal role. Another major disadvantage that limits its widespread applicability is the high cost and the specialized housing that is required (101). The use of animals with humanized immune system may also provide unique insights and fully recapitulate features of IPF (101).

## Domestic Animals

The field of comparative oncology has set the stage for collaborations that utilize spontaneous models of progressive fibrotic lung diseases of mutual interest to veterinary and human medicine. The results of these kinds of studies promise to enhance the understanding of common factors important to disease development in a variety of species and to refine treatments for both humans and animals. Moreover, they may provide insights into unanswered questions involving naturally occurring models of pulmonary fibrosis.

In contrast to the six million dogs and cats that develop cancers, the incidence and prevalence of pulmonary fibrosis in animals is not known (102). West Highland Terriers (Westies), cats, donkeys, and horses develop ILD (102–107). There is limited information on the spectrum of clinical parameters (e.g., radiology) and pathology of these lung diseases leading to the classification of “idiopathic pulmonary fibrosis” being applied to such cases, without using the same strict clinical criteria that have been developed for human IPF. Recent evidence suggests that in contrast to IPF in humans, applying the term “idiopathic” in animals may be premature because of more non-specific features in lung interstitial disease in animals. The American Thoracic Society/European Respiratory Society definition of IPF incorporates histology, radiographic, and clinical course in the definition and the exclusion of other known causes of ILD, including environmental exposures, connective tissue disease, and drug toxicity (108). Further studies using multidisciplinary classification of veterinary lung disease to better characterize the disease in animals will help to define their relation to human disease and their potential role as models to develop treatments for both human and veterinary medical practice.

A study on Westies (109) found that the majority of dogs with IPF showed multifocal areas of accentuated subpleural and peribronchiolar fibrosis with occasional “honeycombing” and profound alveolar epithelial changes, reminiscent of human UIP and not commonly seen in NSIP. Interstitial fibroblastic foci, characteristic of UIP, were not seen in WHWTs with IPF. Progressive fibrosis, with intra-alveolar organizing fibrosis alongside interstitial mature collagen deposition, was present within the more severely affected areas of lung in WHWTs with IPF. Severe pulmonary lesions were seen more commonly in the caudal than in the cranial lung lobes.

A more recent study correlating CT scans and course of disease in Westies found a generalized ground-glass pattern was determined to be a sign of a mild form of canine idiopathic pulmonary fibrosis, whereas mosaic ground-glass and mild honeycombing patterns was identified in moderate and severe forms of the disease (110).

The ubiquitous gammaherpesvirus equine herpesvirus 5 (EHV 5) has been detected in lung tissue from horses that develop progressive pulmonary fibrosis and is now considered to be the likely cause of this disease in these animals (111). The pathology of this disease is distinct from human IPF, demonstrating multiple nodules and is therefore termed equine multinodular pulmonary fibrosis (111). Although the pathology is not the same as IPF, there are striking overlapping features including weight loss and gradual exercise intolerance, accompanied by characteristic radiologic features (111). Temporal heterogeneity or fibroblast foci, hallmarks of human disease, are not present in the disease in horses, though these characteristics have been described in feline pulmonary fibrosis (111, 112). Similar to EBV in humans, which has been associated with IPF, EHV 5 is a ubiquitous subclinical gammaherpesviral infection in horses (113). Considered largely non-pathogenic in the natural host, some strains of EHV5 appear to be pathogenic and capable of inducing lung fibrosis (103). While EHV5 was isolated from horses with spontaneous disease, the virus was not isolated from dead inoculated horses that developed lung fibrosis (111). This model raises interesting questions regarding induction of lung fibrosis by EHV 5 during viral latency versus lytic infection.

For a tabular representation of the overall advantages and disadvantages of each model, see Table 2.

**Table 2:** Pros/cons of animal models for studying pulmonary fibrosis.

Murine models	Pros	Cons
Bleomycin	Early molecular signature most similar to accelerated acute phase of IPF in humans	Patchy, young mice resolve spontaneously unless repeatedly doses

Silica	Good model of lung injury in humans and persistence of fibrotic lesions	Lack of reproducibility, difficult delivery, prolonged time to fibrosis, absence of usual interstitial pneumonia (UIP)-like lesions

Asbestosis	Recapitulates asbestos exposure in human lung fibrosis	Inhalation model requires at least a month for fibrosis to develop. Single intratracheal dose leads to central fibrosis rather than subpleural, unevenly distributed between lungs

Cytokine overexpressing	Ability to dissect downstream signaling events relevant to specific fibrotic-inducing cytokines	Models limited to dissecting specific pathways, rather than recapitulating the complexity of human disease

Fluorescent isothiocyanate	Relatively reproducible and persistent fibrotic phenotypes	Lack representative UIP and inflammatory infiltrates preceding fibrosis

Radiation induced	Results in fibrosis, not pneumonitis if B6 mice are used	Need to wait a long time for development of fibrosis

Familial models	Gave insight on telomere and telomerase gene involvement in IPF	May produce a susceptible phenotype, requiring a second hit

Humanized (NOD/SCID mice)	Can afford insight into role of different fibroblast populations, dissects the contribution of epithelial-fibroblast crosstalk in the absence of immune cells	May not be representative of human disease where immune cells play a role. Expensive and requires specialized housing

**Domestic animals**	**Pros**	**Cons**

Dogs	Usually present in middle to old age. IPF in Westies shares some features of human disease; foci with severe lesions, histological criteria more typical for UIP may be present. Spontaneously develop ILD	The diffuse interstitial lesion, present in all affected Westies, histologically resembles fibrotic NSIP in man

Cats	Anatomy of distal lung similar to humans. UIP-like disease. Spontaneously develop ILD	Strain-dependent

Donkeys	Spontaneously develop ILD	Majority of cases of APF share key pathological features with human pleuroparenchymal fibroelastosis not IPF

Horses	Spontaneously develop ILD. Overlapping features of pulmonary fibrosis including weight loss and characteristic radiologic findings	Pathology not the same as IPF

### Read-Out Assays for Assessment of Fibrotic Injury

Each experimental model presents with its own kinetics of fibrotic injury; however, investigators have applied standard operating procedures for reproducible evaluations of lung fibrosis. In view of the pathologic hallmarks of IPF, appropriate read-out assays include assessment of collagen deposition, alveolar epithelial cell apoptosis, and BALF complemented by survival analysis and respiratory mechanics. These are achieved with the following modalities: (1) histological analysis with Masson trichrome and H&E staining coupled with Aschroft score that quantifies extent of fibrotic changes, (2) hydroxyproline or total collagen content for quantification of lung collagen deposition, (3) TUNEL assay for the identification of apoptotic cells, (4) BALF analysis to assess changes in differential cell count and levels of inflammatory and fibrotic markers, (5) survival analysis with Kaplan–Meier plots, (6) *in vivo* lung function measurements (elastance and compliance) using the Flexi-vent ventilator, and (7) micro-CT imaging which provides state-of-the art multidimensional imaging of the injured lung that is reminiscent of HRCT applied for IPF diagnosis (19).

### Limitations

The past 35 years more than 500 experimental studies have been performed describing therapeutic efficacy of novel compounds in the BLM model. Unfortunately, less than 5% have applied a therapeutic protocol indicated by drug administration at >7 days following BLM challenge (18, 114). Even day 7 in most of the experimental models represents a stage of inflammation or early fibrosis, evidence that comes in contrast to the clinical situation in which treatment is initiated after onset of symptoms and when fibrosis has already been established. Intriguingly, pirfenidone and nintedanib received approval to proceed to clinical trials based on preventive protocols or even therapeutic protocols targeting the inflammatory or the early-fibrotic phase of the BLM model (115–117). In addition, most of the therapeutic compounds have been preclinically tested in young animals while it has been clearly shown that aged mice are more susceptible to fibrotic injury (26), which is in accordance with patients with IPF. Importantly, preclinical efficacy of the majority of anti-fibrotic agents was tested in a single model, majorly the BLM-induced model, and based on histologic end points, such as collagen deposition that are not clinically relevant, at least to the extent of lung function tests or survival analysis. Moreover, many of the therapeutic outcomes were subject to evaluation bias considering that most of the preclinical studies were not blinded and the investigator was aware of the animals’ treatment. Reproducibility issues arising from different experimental settings between different labs could also account for discrepancies in treatment effects and lack of generalizable results. Finally, it is worth mentioning that animal size needs to be balanced with the statistical power needed to generate robust data and that insufficient reporting of experimental animal data or unpublished negative therapeutic results severely hamper the validity of experimental studies.

### Conclusion and Personal View

An animal model is a simple representation of a complex biology system. Critics focusing on the reasons why an animal model cannot fully reproduce human disease are not helpful and do not elicit a solution. The role of an animal model is to recapitulate specific aspects of a disease. Consequently, animal models should be carefully selected, designed, and conducted in order to bridge translational gaps between bench and bedside. Currently, the BLM model of lung fibrosis represents the cheapest, easiest, fastest, most reproducible, and thus most extensively used animal model of IPF; advantages that overcome the handicap of minimal representation of human disease. So far, it has provided us with invaluable insights into IPF pathogenesis, prognosis, and treatment. We recommend the use of the male aged BLM mouse model as the first-line animal model to test safety and efficacy of a therapeutic compound administered during the stage of established fibrosis. Nevertheless, efficacy preclinical studies should be enriched with two or even three animal models including clinically relevant end points such as lung function mechanics, survival analysis as well as biomarkers (28). Collaboration between veterinary and human clinical researchers must be encouraged in order to establish and solidify a common language, and common diagnostic criteria and nomenclature, thus strengthening the opportunity for advancements toward a cure for lung fibrosis in both animal and humans. Humanization of animal models, spontaneous fibroses animals and application of high-throughput “omics” tools may help us improve the clinical translation in the near future.

## Author Contributions

Conception and study oversight: MG, AL, VA, and AT. Drafting manuscript: JT, GR, KW, SJ, and IN. Critical revision of manuscript: MG, AL, VA, and AT. Final approval: all authors.

## Conflict of Interest Statement

The authors declare that the research was conducted in the absence of any commercial or financial relationships that could be construed as a potential conflict of interest.
